# Occurrence Patterns of Lichens on Stumps in Young Managed Forests

**DOI:** 10.1371/journal.pone.0062825

**Published:** 2013-04-24

**Authors:** Måns Svensson, Anders Dahlberg, Thomas Ranius, Göran Thor

**Affiliations:** 1 Department of Ecology, Swedish University of Agricultural Sciences, Uppsala, Sweden; 2 Department of Forest Mycology and Plant Pathology, Swedish University of Agricultural Sciences, Uppsala, Sweden; Umea University, Sweden

## Abstract

The increasing demand for forest-derived bio-fuel may decrease the amount of dead wood and hence also the amount of available substrate for saproxylic ( = dead-wood dependent) organisms. Cut stumps constitute a large portion of dead wood in managed boreal forests. The lichen flora of such stumps has received little interest. Therefore, we investigated which lichens that occur on stumps in young (4–19 years), managed forests and analyzed how species richness and occurrence of individual species were related to stump and stand characteristics. We performed lichen inventories of 576 Norway spruce stumps in 48 forest stands in two study areas in Central Sweden, recording in total 77 lichen species. Of these, 14 were obligately lignicolous, while the remaining were generalists that also grow on bark, soil or rocks. We tested the effect of characteristics reflecting successional stage, microclimate, substrate patch size, and the species pool in the surrounding area on (1) total lichen species richness, (2) species richness of obligately lignicolous lichens and (3) the occurrence of four obligately lignicolous lichen species. The most important variables were stump age, with more species on old stumps, and study area, with similar total species richness but differences in occupancy for individual species. Responses for total lichen species richness and species richness of obligately lignicolous lichens were overall similar, indicating similar ecological requirements of these two groups. Our results indicate that species richness measurements serve as poor proxies for the responses of individual, obligately lignicolous lichen species.

## Introduction

In natural boreal forest ecosystems, dead wood is created through self-thinning and disturbances such as storms, fires and pest outbreaks [1]–[4]. Modern forestry, with forest rotations of less than 100 years, establishment of even-aged monocultures, effective fire suppression, and routine thinning and clear-cutting, has resulted in a sharp decrease in the amounts of coarse dead wood (CWD) [4]–[6]. Consequently, many forest species dependent on CWD have experienced marked declines [4]. In Sweden, for example, about half of the ca 2000 red-listed forest-dwelling species are dependent on CWD [7]. The formation and maintenance of CWD in managed forests are thus among the crucial factors for a successful biodiversity management [3]–[4], [8].

In Fennoscandia, the standard practice since large-scale clear-cutting was introduced in the 1950's has been to extract the stems and leave the top, the branches and the stump. Since the end of the 20^th^ century, increasing energy prices and climate concerns have increased the interest in using these residues as a bio-fuel [9]. Bio-fuel extractions from clear-cuts in Sweden have so far concerned slash (tops and branches), but stumps left after final felling are increasingly being used. A large proportion of all dead wood left after clear-felling consists of stumps [10], with some inventories indicating that up to 80% of the CWD left are in the form of stumps [11]. Research on consequences of large-scale stump harvest for biodiversity is therefore much needed, since today's management recommendations for stump extractions are based on insufficient knowledge of the potential effects [12].

Dead wood is utilized by many different groups of organisms, in particular many species of fungi and insects [4], [13]–[14]. In boreal forests, lichens are a species rich group on wood. Although wood as a lichen substrate has much in common with bark, it does have distinct properties and thus, a number of species specialized on this substrate have evolved. In Fennoscandia, 378 lichen species occur on wood [15]. Of these, 97 are considered obligately lignicolous (i.e. dependent on wood, for other taxa, the term ‘saproxylic’ is often used). The remaining 281 species are known to have a large proportion of their occurrences on other substrates, such as soil or bark [15]. Classifying saproxylic organisms in ecological groups (as for, e.g., beetles, [16]–[17]) has been found to be a fruitful way of furthering our understanding of the biodiversity of dead wood, but will depend on the availability and quality of basic data (data on microclimatic preferences, substrate specificity etc.) on the biology of the organisms concerned. The lack of such data for most lignicolous lichen species have sometimes led to analyses of factors of importance for overall lichen species richness on wood (e.g. [18]), although such an approach usually means that the responses are mainly affected by the occurrence of common, generalist species, as they are usually dominant (cfr. [15], [19]). Drivers of lichen species richness on wood include degree of exposition and degree of decomposition [20]–[22] and substrate age [21]. The properties of adjacent stands may also influence the probability of occurrence of individual, lignicolous lichens [23]. As particularly the populations of obligately lignicolous species could be hypothesized to decrease following a further reduction in CWD, understanding their ecological requirements and distributional patterns are an important step in the direction of a sustainable management practice. The classification of Spribille et al. [15] could be used as a basis for studies focusing on lignicolous lichens.

The aim of this study was to identify which lichens that occur on Norway spruce stumps in young managed forests and to analyze how their occurrence and species richness were influenced by different characteristics reflecting successional stage (i.e. degree of decomposition, stump age), microclimate (bryophyte cover, stump height, shading), substrate patch size (stump surface area), and species pool in the surrounding landscape (age of the stands surrounding clear-cuts). In particular, we were interested in understanding the requirements of obligately lignicolous species. We compared the outcome of three different response variables: (a) total lichen species richness, (b) species richness of obligately lignicolous lichens and (c) occurrence of individual obligately lignicolous lichens. We hypothesized that the response of total species diversity would differ from those of the sub-sets of the obligately lignicolous species. Clear differences among these response variables would imply that greater precision in identifying factors relevant to conservation could be reached by selectively analyzing the more specialized, obligately lignicolous species.

## Materials and Methods

### Study areas

The study was performed in the provinces of Dalarna (Fredriksberg, 60°05′N, 14°05′E, altitude 300–400 m.a.s.l.) and Östergötland (Finspång, 58°48′N, 15°41′E, 60–70 m.a.s.l.) in Central Sweden, in the the southern boreal and the boreo-nemoral zone, respectively [24]. Permission for fieldwork was obtained from the landowners, Bergvik Skog AB (Fredriksberg) and Holmen Skog AB (Finspång). All necessary permits were obtained for the described study, which complied with all relevant regulations. In each province a managed forest landscape of about 150 km^2^ was chosen as a study area. In both areas, >95% is owned by a single commercial forest company and consists of managed coniferous forests of varying ages and forested mires not used for forest production. In both cases, stands older than 110 years constitutes <5% of the area of productive forest. The forests are predominantly monocultures or mixed stands of Norway spruce (*Picea abies*) and Scots pine (*Pinus sylvestris*), with occasional occurrences of deciduous trees of which the most common are aspen (*Populus tremula*) and birch (*Betula pendula* and *B*. *pubescens*). Clear-cutting is typically done at a stand age of 80–100 years. In each region, twelve stands in two age classes (4–7 years and 16–19 years after cutting) were randomly selected using the stand databases of the land owners. Norway spruce stumps younger than 4 years generally have no lichens ([19] and own observations), while stumps older than 20 years generally are in advanced state of decomposition and a high proportion of them no longer serve as a lichen substrate [19]. Stands included in the randomization were Norway spruce dominated (>70%) forests, as this type of stand is the most likely to be harvested for stumps. The other trees present were Scots pine as well as naturally regenerated deciduous trees. In the younger age class, the trees rarely exceeded 1 m in height, while in the older age class, the height ranged between 6–10 m. Stands on islands or more than 2 km from a road (<1% of the stands and <0.5% of the area respectively) were not included.

### Sampling

In every stand, a transect was placed at the longest possible distance through each stand. The start of the transect was 25 m from the edge of the stand. Twelve points were positioned with even distances along the transect. At every such point, the closest Norway spruce stump was chosen, thus resulting in twelve stumps chosen per stand. Stumps included should have a diameter of 20–70 cm. Smaller or larger stumps were rare. Stumps severely damaged during clear-cutting or stumps within 1 m of another stump or a boulder were excluded. Lichen species present on stumps were registered as occurring on the (i) cut surface, (ii) the lateral wood surface or on (iii) bark, if any remained. Most species were determined in the field, but when required, samples were collected to be identified later using microscopy and/or thin-layer chromatography (HPTLC). *Bacidina* sp. could not be determined to species level and possibly represents an undescribed species (S. Ekman, pers. comm. 2012). *Lepraria* species were generally not determined to species, but all samples analyzed (<10% of all records, from both regions) belonged to *L*. *incana*. *Micarea prasina* might include *M*. *byssacea* and/or *M*. *micrococca*, but samples analyzed by thin-layer chromatography (>90% of all records) all belonged to *M*. *prasina* s. str. Four species that are not lichenized or doubtfully lichenized (*Arthonia coronata*, *Arthrorhaphis aeruginosa*, *Mycocalicium subtile*, *Thelocarpon epibolum*) but traditionally are treated by lichenologists have been included. They are hereafter referred to as lichens. Collections by the first author will be deposited in herbarium UPS. Raw data are available from the authors on request. Nomenclature for lichens follows [25]. Field work was carried out during 2009–2010.

### Explanatory variables

For every stump, the height in cm was measured using a measuring tape, using the ground as reference level. The total wooden surface area (cut surface + lateral wood surface) was estimated. Stumps included had a height between 10–80 cm. Shadiness was estimated for every stump using the scale: (1) exposed to direct sunlight, (2) semi-shaded, and (3) never exposed to direct sunlight. Degree of decomposition was estimated according to a scale similar to that of [26]: (1) hard wood, knife penetrating only a few mm, (2) knife penetrating 1–2 cm, (3) knife penetrating 2–5 cm, (4) knife penetrating >5 cm, (5) soft wood, substrate crumbles under pressure. Estimates of decomposition were done six times per stump, including both cut surface and lateral wood surface, but not bark. The final decomposition estimate was the mean of these estimations. Bryophyte cover was estimated as the proportion of each stump covered by bryophytes. We tested the effect of the age of the surrounding stands, by defining buffer zones extending 1 km from the edge of each sampled stand. Since obligately lignicolous species might disperse from both adjacent young stands (where other similar stumps and other types of sun-exposed dead wood occur) or from old forest (which tend to be more rich in wood substrates than younger forests, [4]), the proportion of both forest 0–20 years old and the proportion of forest older than 90 years were calculated for the buffer zones using stand databases and the GIS-software ArcMap 10.

### Statistical analyses

For comparison of the species density between stands of the two age classes in the two regions, sample-based rarefaction curves were used [27], calculated with 50 random re-samplings among samples. For the calculation of the rarefaction curves and species richness estimators, we used EstimateS vers. 8.2.0 [28].

The importance of the explanatory variables for lichen species richness and the occurrence of selected lichen species on individual stumps were evaluated using generalized linear mixed models (GLMM). We selected those lichen species considered obligately lignicolous by [15] that occurred on more than 30 stumps, which were *Micarea denigrata* (39 stumps), *Mycocalicium subtile* (31 stumps), *Xylographa parallela* (118 stumps) and *X*. *vitiligo* (33 stumps). *Cladonia botrytes*, which was considered obligately lignicolous in [15], has subsequently been shown to be only a facultatively lignicolous species ([29] and own observations) and was thus not counted as an obligately lignicolous species in this study. When the response variable was species presence/absence per stump, we used a binomial probability distribution and a logit link function, with stand identity included as a random effect in all models. When the response variable was the number of obligately lignicolous lichen species per stump or the total number of lichen species per stump, we instead used a Poisson distribution and a logarithmic link function. As full models incorporating the estimates of shadiness always failed to converge, this variable was excluded from further analysis. After generating the GLMM, the explanatory variables were standardized to allow for comparisons of their respective effect size [30]–[31]; then, a set of sub-models including all possible combinations of the explanatory variables was generated [32]. The differences (Δi) of the AIC_c_ (Akaike's Information Criterion corrected for small sample sizes) for each sub-model were used to rank the models with a Δi <6 used as a threshold for a model to be considered as having support. The relative variable importance (RVI) was estimated, on a scale of 0 to 1, by summing the AIC_c_ weights across all sub-models in which the variable occurred. Better models have larger AIC_c_ weights and consequently, variables that contribute more to model fit will have a higher RVI. In our analyses, we generally could not find a single best model and the effect sizes are thus given by model-averaged parameter estimates. The precision of these estimates account for model selection uncertainty, which is included in the estimated range of the confidence intervals. To provide values for the goodness-of-fit for the GLMM:s, conditional and marginal R^2^ values were calculated for the full models using the method of [33]. The conditional R^2^ value shows the proportion of the variance in the raw data explained by the full model, including both fixed and random effects, while the marginal R^2^ value shows the proportion of the variance explained by the fixed effects only.

The importance of the explanatory variables at stand level was evaluated using the same statistical analyses as at the stump level. As explanatory variables we used the proportion young (0–20 years) and old (>90 years) forest surrounding the stands, study area, and stump age. We used generalized linear models (GLM), from which model averaging was performed as above. We used a binomial distribution and a logit link function for the individual species response variables (the ratio of stumps with species presence per stand). When the response variable was the total number of lichen species or number of obligately lignicolous lichen species per stand, a Poisson distribution and a logarithmic link function was used.

The GLM:s and GLMM:s were performed in R [34] , using the lme4 package [35]. The standardization of explanatory variables was done using the arm package [36] and the MuMIn package [37] was used for the multimodel inference.

## Results

**Figure 1:**
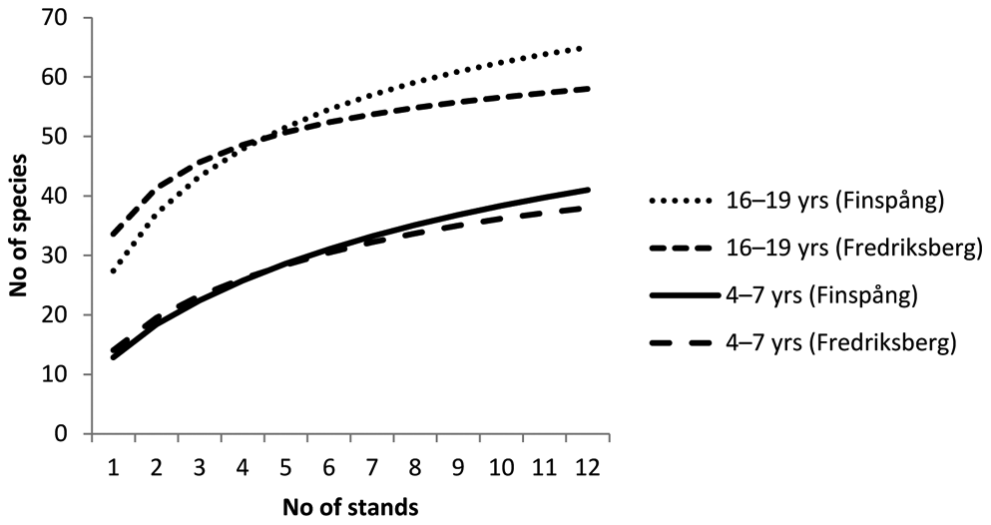
Sample based rarefaction curves comparing species density between young and old stumps (on young and old clear-cuts, respectively) at Finspång and Fredriksberg. In each stand, 12 stumps were surveyed. The 95% confidence limits have been excluded for clarity, but did not overlap between young and old stumps.

In total, 576 spruce stumps were included in the inventory (Table 1). 77 lichen species were found, of which 14 are considered obligately lignicolous (Table S1). Most of the species occurred on all three parts of the stumps, but three species where only found on cut surfaces, 10 only on lateral wood surfaces and two only on bark. With the exception of a single find of *Absconditella delutula* (VU, [5]), no red-listed species was found. The sample-based rarefaction curves (Fig. 1) have all started to level out, indicating that most of the lichen species present were recorded. Model statistics are included in Model Output S1.

**Table 1:** Average values (± SE) for variables recorded in survey of Norway spruce stumps in two study areas in southern Sweden.

	Finspång		Fredriksberg	
	Stump age		Stump age	
	3–7 yrs	16–19 yrs	3–7 yrs	16–19 yrs
Bryophyte cover (%)	7.7 (1.1)	32.1 (1.8)	11.2 (0.8)	35.3 (2.0)
Decomposition (index 1–5)	1, 3	3, 5	1, 3	3, 5
Stump height (cm)	34 (0.8)	30.2 (0.8)	31.3 (0.8)	33 (0.7)
Wooden surface area (dm^2^)	26.7 (1.6)	24 (1.2)	19.4 (1.0)	25.0 (1.4)
Prop. forest 0–20 yrs in buffer zone (%)	17 (2.3)	19.4 (1.9)	26.9 (3.2)	23.9 (3.1)
Prop. forest >90 yrs in buffer zone (%)	5.2 (0.6)	7.8 (1.8)	8.7 (1.5)	14.9 (2.6)
No of species/stump	4.6 (0.2)	8.2 (0.4)	4.8 (0.2)	13 (0.9)
Stumps with species records (%)	96.5	93.7	97.9	95.8
Stumps with obligately lignicolous lichens (%)	4.9	34.5	8.3	77.8

For decomposition, maximum and minimum values are given instead of average. 12 stands and 144 stumps were surveyed in each category.

### Effects of stump-level variables

**Figure 2:**
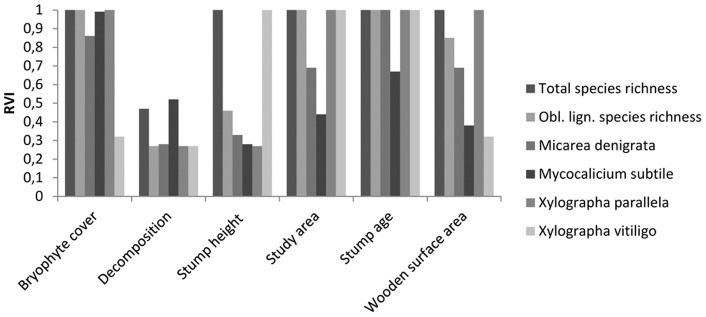
Relative importance of stump-level explanatory variables (RVI) for total species richness, obligately lignicolous species richness, and the occurrence of four individual lignicolous lichen species on Norway spruce stumps.

**Figure 3:**
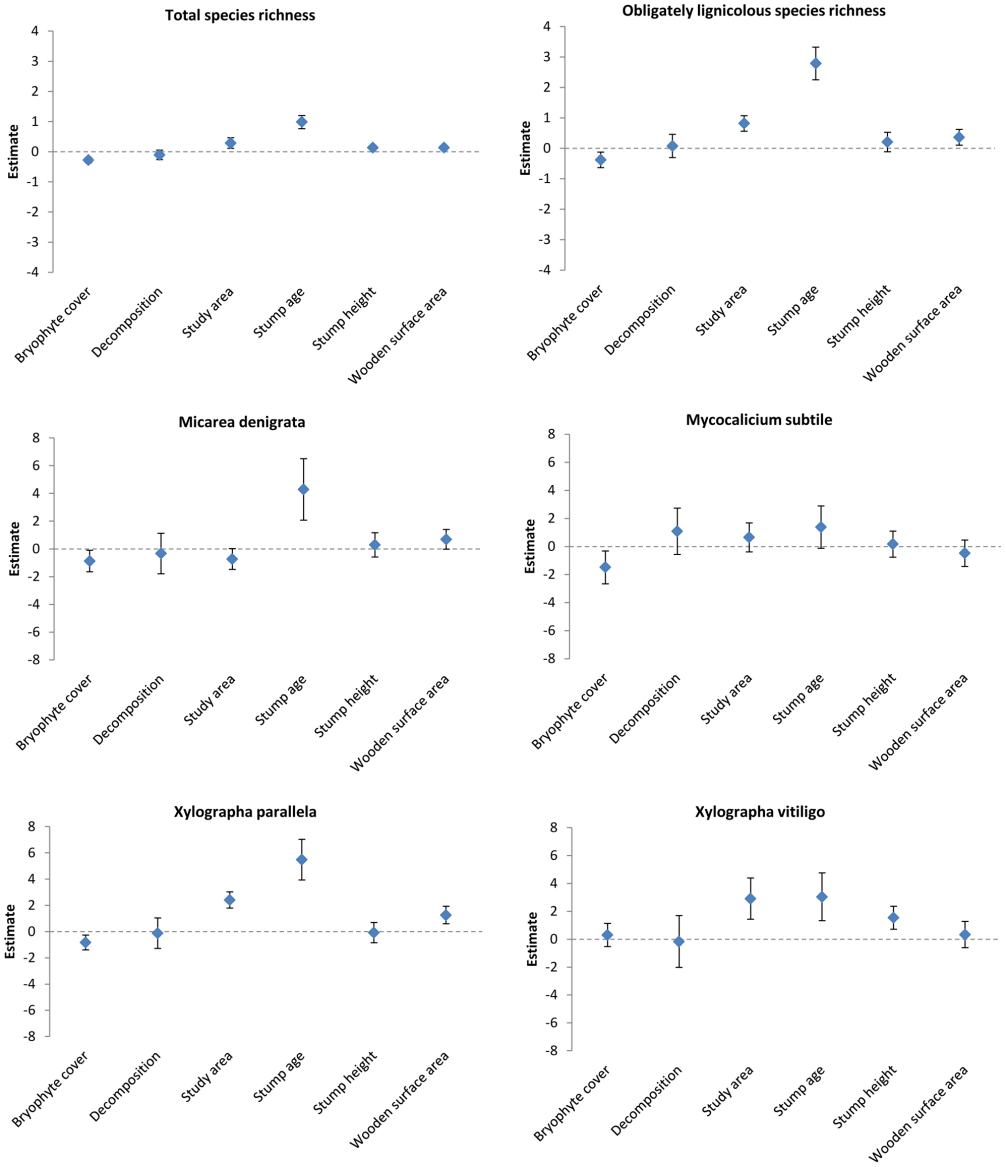
Model-averaged parameter estimates and 95% confidence intervals for total lichen species richness, obligately lignicolous species richness and presence of four lichen species on Norway spruce stumps. Note the difference in scales.

Generally, across all analyses stump age and study area had the largest effect sizes (Figs. 2 & 3). The number of explanatory variables with clear effects (i.e. confidence intervals not overlapping zero) was five for total species richness, four for obligately lignicolous species richness and ranging between one to four between the four individual lichen species (Fig. 3). Conversely, effect sizes were low (often close to zero) for total species richness, progressively larger for obligately lignicolous species richness and usually substantially larger for individual, obligately lignicolous species (Fig. 3). There was a positive effect of stump age both on total species richness, species richness of obligately lignicolous lichens, and the probability of finding individual, obligately lignicolous species. The results indicated a positive relationship between species richness per stump, both total and obligately lignicolous, and the northern study area (Fredriksberg). Stump height had a positive effect on total species richness and on one individual species (*Xylographa vitiligo*). Wooden surface area had a positive effect on total species richness, obligately lignicolous species richness and on one individual species (*X*. *parallela*). Increasing bryophyte cover generally had a negative effect on the response variables. We only found weak support for an effect of decomposition, which had the overall lowest relative variable importance and had confidence intervals always encompassing zero (Figs. 2 & 3).

### Effects of stand-level variables

**Figure 4:**
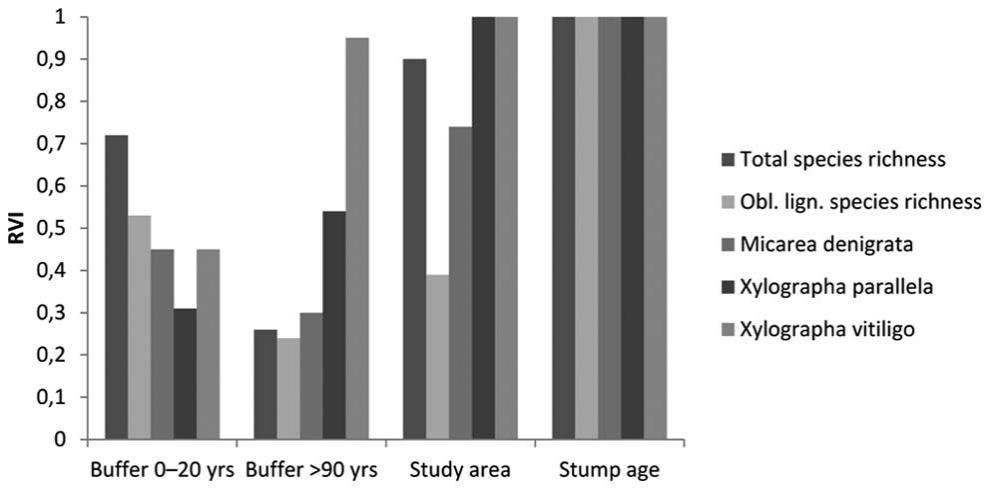
Relative importance of stand-level explanatory variables (RVI) for total lichen species richness, obligately lignicolous species richness and occupancy of three obligately lignicolous lichen species on Norway spruce stumps in 48 young managed forest stands.

**Figure 5:**
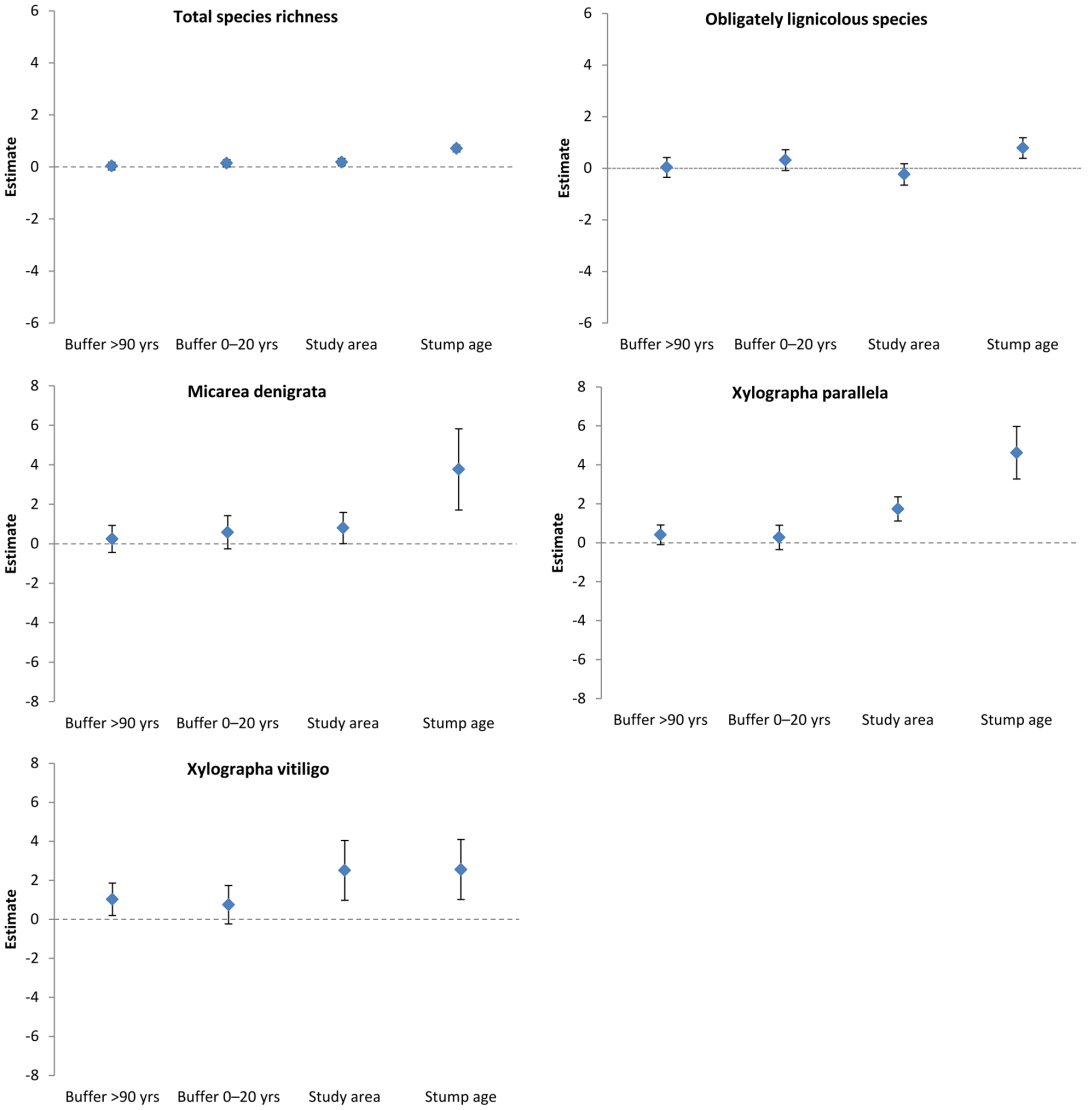
Model-averaged parameter estimates and 95% confidence intervals for total lichen species richness, obligately lignicolous species richness and presence of three lichen species on Norway spruce stumps in 48 young managed forest stands. Note the difference in scales.

Stump age was the most important variable on the stand-level, followed by study area (Fig. 4). The proportion of young (0–20 years) forest surrounding the stand had a positive effect on total species richness per stand (Fig. 5). Proportion old (>90 years old) forest surrounding the stand had a clear effect on one individual species (*X*. *vitiligo*, Fig. 5). For *Mycocalicium subtile*, the null model was included in the set of candidate models. Results for this species were therefore judged as unreliable and are not shown.

## Discussion

### Species composition

The majority of the species found on stumps in this study (82%) are species that may also be found on other substrates apart from wood. Especially, several of the species of *Cladonia* (the majority of which are generalists) were often dominant and, together with bryophytes, they reduce the amount of available substrate for obligately lignicolous lichens. This is illustrated by the fact that the most common obligately lignicolous lichen (*Xylographa parallela*) found in this study occurred on 20% of the stumps, while the second most common (*Micarea denigrata*) occurred on 7%. A similar study that examined the lichen flora on Norway spruce stumps in another province in Central Sweden [19] reported 52 lichen species from the cut surfaces, with the dominant, generalist species being mainly the same as those found by us in our study, with no red-listed species found. There are, however, also differences, as [19] reported 15 species not found by us (including one obligately lignicolous, *Pycnora sorophora*) from cut surfaces. Moreover, on the cut surfaces of our study, we found 17 species (including two obligately lignicolous, *Absconditella delutula* and *Catillaria erysiboides*) not reported by them (not counting species treated collectively in [19] that were kept separate by us). However, as the majority of these aberrant species were singeltons or rare, these differences may just bean effect of limited sampling efforts.

### Occurrence patterns of lignicolous lichens

We found the lichen assemblages on Norway spruce stumps dominated by generalist species, a result similar to other studies on dead wood in young managed forests (e.g. [19]). Thus, when using total species richness as a response variable, it was possible that the responses of obligately lignicolous lichens could have been obscured by the responses of the more common generalists. However, our hypothesis of a clear difference in response between total species richness and obligately lignicolous species richness received no support. Instead, the main difference in response between total species richness and the obligately lignicolous species richness are differences in effect sizes, as the variables with the highest RVI were the same in both cases. Cut stumps left after clear-cutting is a short-lived, artificial substrate, to which no species have evolved. Lichen species growing on such stumps are possibly opportunistic pioneer species with similar ecological requirements. In such a case, the responses of generalists and specialists would be similar, which could explain our results. However, in the case of the individual, obligately lignicolous species, the responses were more differentiated. Thus, using the responses of total species richness or species richness of obligately lignicolous lichens seems to be a poor way of understanding the ecology of individual, obligately lignicolous species. This study included a low diversity of wood qualities and micro-climates. We hypothesize that if wood in more heterogenoeus habitats (e.g. old-growth forest or forest-mire mosaics) would be sampled, the responses of total species richness and obligately lignicolous species richness would show more marked differences. Such habitats could include obligately lignicolous species with very specific requirements, and those species might only rarely or not at all occur on clear-cuts or in young managed forest stands. 83 of the obligately lignicolous lichen species listed by [15] for Fennoscandia were not found by us. Several of these species could have more specific habitat requirements regarding, e.g., different kinds of wood and other micro-climates than those found in the stands included in this study.

### Importance of stump characteristics

Study area was generally an important variable, which for the individual species analyzed simply means a higher abundance in one of the two study areas. In this study, study area is a complex variable and might include effects of, e.g., climate, forest history and differences in management. Stump age was also an important variable in all analyses, reflecting the fact that as colonisation events increase over time, both the total number of species and the number of obligately lignicolous species increases. Increases in stump height have earlier been shown to increase total lichen species richness [21] – a result which this study confirms, though no similar effect was detected for obligately lignicolous species richness or individual obligately lignicolous species (with the exception of *Xylographa vitiligo*). Stumps of greater height will experience a drier micro-climate, which might favour desiccation-tolerant lichens over competing bryophytes and thus increase the number of lichen species [21], [38]. Although a management practice that increases the average height of stumps left after clear-felling could possibly enhance the total species richness of lichens, this study gives little support that obligately lignicolous lichen species would benefit from such a practice.

Wooden surface area had an effect on total species richness, obligately lignicolous species richness and on one individual species (*X*. *parallela*). Increases in the wooden surface area could possibly increase species diversity by a species-area effect. Also, a large stump decomposes more slowly [39], and it would thus serve as a lichen substrate for a longer time. However, effect size was low and therefore, a management practice that retain the largest stumps would probably only have a weak positive effect on obligately lignicolous lichen species.

Bryophyte cover generally had a negative effect on lichens. Bryophytes are often the main competitors with lichens on wood and generally more shade-tolerant [4], [21]. In this study, bryophyte cover also to some extent serves as a proxy for shade. In contrast to earlier studies [20]–[22], we found no support for an effect of decomposition on the responses of both the species richness measurements and the responses of individual lichen species. Decomposition of wood is a complex process and depending on, e.g., exposure, moisture, composition of the fungal community and tree species, wood with very different qualities may be produced [4]. The rather low effect of decomposition in our study is likely due to the homogeneous nature of the stumps, generating a strong correlation between stump age and decomposition (i.e. stumps of even-aged trees cut at the same time and subject to similar environmental conditions). In forests holding a greater diversity of wood-types, different decompositional stages might contribute more to the lichen diversity.

### Importance of the age of the surrounding forest

An increasing proportion of young (0–20 years) forest surrounding the investigated stands had a positive effect on total lichen species richness, which indicate that adjacent young stands may serve as dispersal sources for several of the species found on stumps on clear-cuts. The effect size was small, however. We found no support for an effect of the age of the surrounding forest on any of our individual obligate lignicoles, with the exception of *Xylographa vitiligo*. For this species, the probability of occurrence increased with higher amounts of older forest surrounding the stand. This is the only species of the three individual species analyzed at stand-level which mainly reproduces vegetatively by producing soredia. Such relatively large propagules have been suggested to be mainly involved in short-distance dispersal [40].

### Conclusions

It is unlikely that any lignicolous lichen species has an obligate relationship with cut stumps, as such stumps are not a natural substrate and have only in recent times appeared in large quantities. However, since natural CWD is rare in today's managed forests, cut stumps may have become an important substrate for some lignicolous species. An evaluation of the impact on the populations of obligately lignicolous lichens of further decreases of CWD is only possible if data on their occurrence on other types of dead wood in the landscape is available.

In line with earlier studies, this study showed that the majority of species found on spruce stumps in young managed forests are common, generalist species, while a smaller proportion (typically 10–18%) consists of obligately lignicolous species. We found indications that the obligately lignicolous species found on clear-cuts could have similar ecological requirements as the generalist species. This pattern could be typical also of lichen assemblages on natural CWD in more heterogeneous forest types than young managed forests. If the aim is to understand the ecological requirements of the obligately lignicolous lichen species in such habitats, the value of total species richness as a response variable is unclear and needs to be further evaluated.

## Supporting Information

Table S1 Frequency of of 77 lichen species on 576 stumps of Norway spruce in two study areas in Central Sweden (DOC).
(DOCX)

Model Output S1 Output from multimodel inference in R, including AIC-rankings of candidate models and R^2^ values for full models (DOC).
(DOCX)
