# Supplementary material for: Occurrence Patterns of Lichens on Stumps in Young Managed Forests
Source: PLoS One. 2013 Apr 24;8(4):e62825. doi: 10.1371/journal.pone.0062825 (PMC3634766; doi:10.1371/journal.pone.0062825)
Supplement: Table S1 — Frequency of of 77 lichen species on 576 stumps of Norway spruce in two study areas in Central Sweden (DOC). (DOCX) [file pone.0062825.s001.docx]

**Table S1. Frequency of occurrence of lichen species on 576 stumps of Norway spruce in two regions in Central Sweden.**

|  | | **Finspång** | | | | | | | **Fredriksberg** | | | | | | |
| --- | --- | --- | --- | --- | --- | --- | --- | --- | --- | --- | --- | --- | --- | --- | --- |
|  | | **4–7 yrs old stumps** | | | **16–19 yrs old stumps** | | | | **4–7 year old stumps** | | | | **16–19 yrs old stumps** | | |
| **Species** | **cut surface** | **lateral surface** | **bark** | **cut surface** | | **lateral surface** | **bark** | **cut surface** | | **lateral surface** | **bark** | **cut surface** | | **lateral surface** | **bark** |
| *Absconditella delutula** | 0.7 (0.7) | - | - | - | | - | - | - | | - | - | - | | - | - |
| *Absconditella lignicola** | - | - | - | 1.4 (1.0) | | 3.5 (1.5) | 0.7 (0.7) | - | | - | - | 4.9 (1.8) | | 2.1 (1.2) | - |
| *Arthonia coronata* | - | - | - | 2.1 (1.2) | | 3.5 (1.5) | 0.7 (0.7) | - | | - | 1.4 (1.0) | 4.9 (1.8) | | 13.2 (2.8) | 7.6 (2.2) |
| *Arthrorhaphis aeruginosa* | - | - | - | 6.3 (2.0) | | 4.2 (1.7) | - | - | | - | - | 0.7 (0.7) | | - | - |
| *Bacidina* sp. | 4.9 (1.8) | 0.7 (0.7) | 0.7 (0.7) | 1.4 (1.0) | | - | - | 16.7 (3.1) | | 2.1 (1.2) | 2.1 (1.2) | - | | 4.9 (1.8) | 4.2 (1.7) |
| *Bryoria capillaris* | - | - | - | - | | 0.7 (0.7) | - | - | | - | - | - | | - | - |
| *Calicium glaucellum* | - | - | - | - | | 10.4 (2.6) | 0.7 (0.7) | - | | - | - | - | | 7.6 (2.2) | - |
| *Calicium salicinum* | - | - | - | - | | 0.7 (0.7) | - | - | | - | - | - | | 0.7 (0.7) | - |
| *Calicium trabinellum** | - | - | - | - | | - | - | - | | - | - | - | | 2.8 (1.4) | - |
| *Catillaria ameibospora* | - | - | - | - | | - | - | - | | - | - | - | | 0.7 (0.7) | - |
| *Catillaria erysiboides** | - | - | - | 0.7 (0.7) | | - | - | - | | - | - | - | | - | - |
| *Catillaria nigroclavata* | - | - | - | - | | 0.7 (0.7) | - | - | | - | - | - | | - | - |
| *Catinaria atropurpurea* | - | - | - | - | | - | - | - | | - | - | 0.7 (0.7) | | 2.8 (1.4) | - |
| *Cetraria islandica* | - | - | - | - | | - | - | - | | - | - | 8.3 (2.3) | | 2.1 (1.2) | 0.7 (0.7) |
| *Chaenotheca brunneola** | - | - | - | - | | 1.4 (1.0) | - | - | | - | - | - | | - | - |
| *Chaenotheca chrysocephala* | - | - | 0.7 (0.7) | - | | - | - | - | | - | - | - | | - | - |
| *Cladonia arbuscula* | - | - | 0.7 (0.7) | 26.4 (3.7) | | 11.1 (2.6) | 2.1 (1.2) | 1.4 (1.0) | | 0.7 (0.7) | - | 76.4 (3.6) | | 27.1 (3.7) | 8.3 (2.3) |
| *Cladonia botrytes** | 2.1 (1.2) | - | - | 22.9 (3.5) | | 11.8 (2.7) | 2.1 (1.2) | 1.4 (1.0) | | 1.4 (1.0) | - | 55.6 (4.2) | | 37.5 (4.0) | 8.3 (2.3) |
| *Cladonia carneola* | - | - | - | 0.7 (0.7) | | 1.4 (1.0) | 2.1 (1.2) | - | | - | 2.8 (1.4) | 15.3 (3.0) | | 9.7 (2.5) | 2.8 (1.4) |
| *Cladonia cenotea* | - | - | 7.0 (2.1) | 38.2 (4.0) | | 20.8 (3.4) | 2.8 (1.4) | - | | 0.7 (0.7) | 21.5 (3.4) | 70.8 (3.8) | | 59.0 (4.1) | 31.9 (3.9) |
| *Cladonia chlorophaea* | - | - | - | 6.9 (2.1) | | 4.2 (1.7) | 2.1 (1.2) | - | | - | - | 1.4 (1.0) | | 1.4 (1.0) | - |
| *Cladonia coniocraea* | 9.0 (2.4) | 4.9 (1.8) | 67.4 (3.4) | 57.6 (4.1) | | 58.3 (4.1) | 11.1 (26.3) | 18.8 (3.3) | | 3.5 (1.5) | 75.0 (3.6) | 36.1 (4.0) | | 51.4 (4.2) | - |
| *Cladonia cornuta* | - | - | 0.7 (0.7) | 1.4 (1.0) | | 2.8 (1.4) | 0.7 (0.7) | - | | - | - | 23.6 (3.6) | | 1.4 (1.0) | - |
| *Cladonia crispata* | - | - | - | - | | 0.7 (0.7) | - | - | | - | - | 24.3 (3.6) | | 2.8 (1.4) | - |
| *Cladonia deformis* | - | - | - | 2.1 (1.2) | | 0.7 (0.7) | 2.8 (1.4) | - | | - | 2.1 (1.2) | 13.9 (2.9) | | 4.9 (1.8) | - |
| *Cladonia digitata* | - | 4.9 (1.8) | 45.8 (4.2) | 18.8 (3.3) | | 41.7 (4.2) | 14.6 (3.0) | - | | 0.7 (0.7) | 35.4 (4.0) | 19.4 (3.3) | | 45.1 (4.2) | - |
| *Cladonia fimbriata* | 11.8 (2.4) | 3.5 (1.5) | 64.6 (4.0) | 54.2 (4.2) | | 62.5 (4.0) | - | 6.3 (2.0) | | 4.2 (1.7) | 68.1 (3.9) | 41.0 (4.1) | | 52.1 (4.2) | - |
| *Cladonia floerkeana* | - | - | 0.7 (0.7) | 1.4 (1.0) | | 0.7 (0.7) | - | - | | - | - | - | | - | - |
| *Cladonia furcata* | - | - | - | 5.6 (1.9) | | 5.6 (1.9) | 2.1 (1.2) | - | | - | - | 2.1 (1.2) | | 2.1 (1.2) | - |
| *Cladonia gracilis* | - | - | - | - | | 0.7 (0.7) | - | - | | - | 0.7 (0.7) | 1.4 (1.0) | | 1.4 (1.0) | - |
| *Cladonia macilenta** | - | - | - | - | | - | - | - | | - | - | 7.6 (2.2) | | 1.4 (1.0) | - |
| *Cladonia norvegica* | - | 0.7 (0.7) | - | 0.7 (0.7) | | 2.8 (1.4) | - | - | | - | 0.7 (0.7) | - | | 2.1 (1.2) | - |
| *Cladonia ochrochlora* | - | - | - | 2.1 (1.2) | | 0.7 (0.7) | 0.7 (0.7) | - | | 0.7 (0.7) | 13.9 (2.9) | 20.1 (3.4) | | 13.2 (2.8) | - |
| *Cladonia pyxidata* | - | - | 4.2 (1.7) | 7.6 (2.2) | | 5.6 (1.9) | 1.4 (1.0) | - | | - | 2.8 (1.4) | 6.3 (2.0) | | 4.9 (1.8) | 5.6 (1.9) |
| *Cladonia rangiferina* | 4.9 (1.8) | 0.7 (0.7) | 4.9 (1.8) | 52.8 (4.2) | | 35.4 (4.0) | 10.4 (2.6) | 2.8 (1.4) | | 0.7 (0.7) | 0.7 (0.7) | 78.5 (3.4) | | 37.5 (4.0) | 15.3 (3.0) |
| *Cladonia squamosa* | - | - | 1.4 (1.0) | 12.5 (2.8) | | 16.0 (3.1) | 6.3 (2.0) | - | | - | 2.8 (1.4) | 10.4 (2.6) | | 9.7 (2.5) | 6.3 (2.0) |
| *Cladonia stellaris* | - | - | - | 2.1 (1.2) | | 0.7 (0.7) | - | - | | - | - | 2.1 (1.2) | | 0.7 (0.7) | - |
| *Cladonia stygia* | - | 0.7 (0.7) | - | - | | - | - | - | | - | - | 2.1 (1.2) | | 0.7 (0.7) | - |
| *Cladonia sulphurina* | - | - | - | 1.4 (1.0) | | 0.7 (0.7) | - | - | | - | 4.9 (1.8) | 29.9 (3.8) | | 18.1 (3.2) | 5.6 (1.9) |
| *Coenogonium pineti* | - | - | 2.8 (1.4) | 0.7 (0.7) | | 1.4 (1.0) | 2.1 (1.2) | - | | - | 1.4 (1.0) | - | | - | - |
| *Hypocenomyce scalaris* | - | - | 2.8 (1.4) | 0.7 (0.7) | | 4.2 (1.7) | 1.4 (1.0) | 0.7 (0.7) | | - | 0.7 (0.7) | - | | 1.4 (1.0) | 1.4 (1.0) |
| *Hypogymnia physodes* | 16.0 (3.1) | 9.0 (2.4) | 18.0 (3.2) | 16.7 (3.1) | | 25.0 (3.6) | 1.4 (1.0) | 10.4 (2.6) | | 2.8 (1.4) | 19.4 (3.3) | 15.3 (3.0) | | 13.2 (2.8) | 1.4 (1.0) |
| *Lecanora saligna** | 0.7 (0.7) | - | - | 0.7 (0.7) | | 6.9 (2.1) | - | - | | - | - | - | | 2.8 (1.4) | - |
| *Lecanora varia* | - | - | - | - | | 1.4 (1.0) | - | - | | - | - | - | | - | - |
| *Lecidea nylanderi* | - | - | 8.3 (2.3) | - | | 16.0 (3.1) | 2.1 (1.2) | - | | - | 4.9 (1.8) | - | | 15.3 (3.0) | 0.7 (0.7) |
| *Lecidea turgidula* | - | 1.4 (1.0) | 0.7 (0.7) | 1.4 (1.0) | | 20.1 (3.4) | - | - | | - | - | - | | 29.2 (3.8) | 1.4 (1.0) |
| *Lepraria* sp*.* | - | - | 76.4 (3.6) | - | | 22.5 (3.9) | 25.7 (3.7) | - | | 0.7 (0.7) | 47.2 (4.2) | - | | 9.0 (2.4) | 24.3 (3.6) |
| *Loxospora elatina* | - | - | 0.7 (0.7) | - | | 0.7 (0.7) | 0.7 (0.7) | - | | - | - | - | | - | - |
| *Micarea denigrata** | 0.7 (0.7) | 0.7 (0.7) | - | 6.3 (2.0) | | 13.2 (2.8) | 0.7 (0.7) | - | | - | - | 0.7 (0.7) | | 9.0 (2.4) | - |
| *Micarea globulosella* | - | - | - | - | | - | - | - | | - | 0.7 (0.7) | - | | - | - |
| *Micarea melaena* | - | - | - | - | | 0.7 (0.7) | - | - | | - | - | - | | - | - |
| *Micarea misella** | 1.4 (1.0) | - | - | - | | 0.7 (0.7) | - | - | | - | - | - | | 4.2 (1.7) | - |
| *Micarea prasina* | 0.7 (0.7) | - | 6.9 (2.1) | 3.5 (1.5) | | 3.5 (1.5) | 2.8 (1.4) | - | | - | 4.2 (1.7) | 0.7 (0.7) | | 0.7 (0.7) | - |
| *Mycoblastus sanguinarius* | - | - | - | - | | - | - | - | | - | - | - | | - | 0.7 (0.7) |
| *Mycocalicium subtile** | - | 2.8 (1.4) | - | - | | 5.6 (1.9) | 0.7 (0.7) | - | | 4.2 (1.7) | - | - | | 9.0 (2.4) | - |
| *Ochrolechia microstictoides* | - | - | 2.8 (1.4) | 0.7 (0.7) | | 3.5 (1.5) | 0.7 (0.7) | - | | - | - | 0.7 (0.7) | | 4.9 (1.8) | 0.7 (0.7) |
| *Parmelia saxatilis* | - | - | - | 0.7 (0.7) | | 1.4 (1.0) | - | - | | - | - | 1.4 (1.0) | | 0.7 (0.7) | - |
| *Parmelia sulcata* | - | - | - | 1.4 (1.0) | | 3.5 (1.5) | - | - | | - | - | 0.7 (0.7) | | 0.7 (0.7) | - |
| *Parmeliopsis ambigua* | - | 1.4 (1.0) | 11.1 (2.1) | 8.3 (2.3) | | 16.0 (3.1) | 2.1 (1.2) | 17.4 (3.2) | | 2.1 (1.2) | 54.2 (4.2) | 53.5 (4.2) | | 61.1 (4.1) | 16.0 (3.1) |
| *Parmeliopsis hyperopta* | - | - | 2.0 (1.1) | - | | 5.6 (1.9) | - | 0.7 (0.7) | | - | 13.9 (2.9) | 32.6 (3.9) | | 45.1 (4.2) | 8.3 (2.3) |
| *Peltigera canina* | - | - | - | 2.1 (1.2) | | 0.7 (0.7) | - | - | | - | - | - | | - | - |
| *Placynthiella icmalea* | 9.7 (2.5) | 6.3 (2.0) | 40.2 (4.1) | 22.9 (3.5) | | 29.2 (3.8) | 4.9 (1.8) | 2.1 (1.2) | | 1.4 (1.0) | 6.9 (2.1) | 17.3 (3.2) | | 23.6 (3.6) | 6.3 (2.0) |
| *Placynthiella uliginosa* | - | - | 1.3 (0.9) | 0.7 (0.7) | | 0.7 (0.7) | - | - | | - | 1.4 (1.0) | 0.7 (0.7) | | - | 0.7 (0.7) |
| *Platismatia glauca* | - | - | - | 0.7 (0.7) | | 2.1 (1.2) | - | - | | - | - | 1.4 (1.0) | | 1.4 (1.0) | - |
| *Puttea caesia* (*syn. *Lecidea symmictella*) | - | - | - | 2.8 (1.4) | | 1.4 (1.0) | - | - | | - | - | 4.9 (1.8) | | 6.3 (2.0) | - |
| *Puttea exsequens** | - | - | - | 3.5 (1.5) | | 1.4 (1.0) | - | 0.7 (0.7) | | - | - | 0.7 (0.7) | | 0.7 (0.7) | - |
| *Puttea margaritella* | - | - | 0.7 (0.7) | - | | 0.7 (0.7) | - | - | | - | - | 0.7 (0.7) | | - | 2.1 (1.2) |
| *Sarcosagium campestre* | - | - | - | - | | 0.7 (0.7) | - | - | | - | - | - | | - | - |
| *Steinia geophana* | 1.4 (1.0) | - | - | 1.4 (1.0) | | - | - | - | | - | - | 2.1 (1.2) | | - | - |
| *Thelocarpon epibolum* | 4.2 (1.7) | - | - | 2.8 (1.4) | | - | - | 1.4 (1.0) | | - | - | - | | - | - |
| *Trapeliopsis flexuosa* | 20.1 (3.4) | 5.6 (1.9) | 24.3 (3.5) | - | | 3.5 (1.5) | 0.7 (0.7) | 6.2 (3.0) | | 1.4 (1.0) | 3.5 (1.5) | 27.8 (3.7) | | 5.6 (1.9) | - |
| *Trapeliopsis granulosa* | - | 1.4 (1.0) | - | 20.8 (3.4) | | 25.7 (3.7) | 4.2 (1.7) | 0.7 (0.7) | | - | - | - | | 22.9 (3.5) | 9.0 (2.4) |
| *Tuckermanopsis chlorophylla* | 1.4 (1.0) | - | 1.4 (0.9) | 0.7 (0.7) | | 2.8 (1.4) | - | - | | - | - | - | | - | - |
| *Violella fucata* | - | 0.7 (0.7) | 0.7 (0.7) | - | | - | - | - | | - | 0.7 (0.7) | - | | - | - |
| *Vulpicida pinastri* | - | 0.7 (0.7) | 0.7 (0.7) | 8.3 (2.3) | | 16.7 (3.1) | - | 10.4 (2.6) | | 0.7 (0.7) | 16.7 (3.1) | 29.9 (3.7) | | 44.4 (4.2) | 8.3 (2.3) |
| *Xylographa parallela** | - | 0.7 (0.7) | - | 0.7 (0.7) | | 16.0 (3.1) | - | 0.7 (0.7) | | - | - | 17.4 (3.2) | | 63.2 (4.0) | - |
| *Xylographa vitiligo** | - | - | - | - | | 1.4 (1.0) | - | - | | - | 1.4 (1.0) | 0.7 (0.7) | | 19.4 (3.3) | 0.7 (0.7) |

Observations of lichen species on each stump are reported separately for cut wooden surface, lateral wooden side and bark and

as mean percentage (± SE) of stumps with occurrence. * denotes species that are obligately lignicolous according to [15].
