# Supplementary material for: Occurrence Patterns of Lichens on Stumps in Young Managed Forests
Source: PLoS One. 2013 Apr 24;8(4):e62825. doi: 10.1371/journal.pone.0062825 (PMC3634766; doi:10.1371/journal.pone.0062825)
Supplement: Model Output S1 — Output from multimodel inference in R, including AIC-rankings of candidate models and R2 values for full models (DOC). (DOCX) [file pone.0062825.s002.docx]

**Supplementary material: Models used in multimodel inference**

**Contains: (A) Table of conditional and marginal R^2^ values for stump-level GLMM:s**

**(B) Stump-level GLMM:s – output from multimodel inference in R**

**(C) Table of explained deviance (‘pseudo-R^2^’) for stand-level GLM:s**

**(D) Stand-level GLM:s – output from multimodel inference in R**

**(A) Table of conditional and marginal R^2^ values for stump-level GLMM:s**

**Values are given for full (global) models**

| **Response variable** | **Total lichen species richness** | **Species richness of OLL** | **Micarea denigrata** | **Mycocalicium subtile** | **Xylographa parallela** | **Xylographa vitiligo** |
| --- | --- | --- | --- | --- | --- | --- |
| **Marginal R^2^** | **0.903** | **0.529** | **0.619** | **0.091** | **0.737** | **0.563** |
| **Conditional R^2^** | **0.938** | **0.529** | **0.621** | **0.305** | **0.737** | **0.563** |

**(B) Stump-level GLMM:s – output from multimodel inference in R**

Explanatory variables

<name of expl.var in this document> = <name of expl.var in main paper>

age2 = Stump age

region = Study area

bryocov = Bryophyte cover

vedarea = Wooden surface area

height = Height

decomp = Decomposition

Formulas used:

modell = lmer(<response> ~ age2 + height + region + decomp + vedarea + bryocov + (1|site), family = binomial)

modell = lmer(<response> ~ age2 + height + region + decomp + vedarea + bryocov + (1|site), family = poisson)

**Response variable: Total lichen species richness**

Component models:

df logLik AICc Delta Weight

12356 7 -480.13 974.47 0.00 0.53

123456 8 -479.24 974.73 0.26 0.47

Term codes:

c.age2 c.region z.bryocov z.decomp z.height z.vedarea

1 2 3 4 5 6

Model-averaged coefficients:

Estimate Std. Error z value Pr(>|z|)

(Intercept) 1.89339 0.04397 43.060 < 2e-16 ***

c.age2 0.98486 0.11134 8.845 < 2e-16 ***

c.region 0.28848 0.08789 3.282 0.001030 **

z.bryocov -0.27831 0.04253 6.543 < 2e-16 ***

z.height 0.13371 0.04036 3.313 0.000923 ***

z.vedarea 0.13640 0.03967 3.438 0.000586 ***

z.decomp -0.10646 0.07911 1.346 0.178390

---

Signif. codes: 0 ‘***’ 0.001 ‘**’ 0.01 ‘*’ 0.05 ‘.’ 0.1 ‘ ’ 1

Relative variable importance:

c.age2 c.region z.bryocov z.height z.vedarea z.decomp

1.00 1.00 1.00 1.00 1.00 0.47

2.5 % 97.5 %

(Intercept) 1.80720600 1.9795679

c.age2 0.76663467 1.2030803

c.region 0.11621818 0.4607504

z.bryocov -0.36167251 -0.1949428

z.height 0.05460910 0.2128099

z.vedarea 0.05864215 0.2141624

z.decomp -0.26150260 0.0485901

**Response variable: Species richness of obligately lignicolous lichens**

Component models:

df logLik AICc Delta Weight

1236 6 -200.98 414.11 0.00 0.40

12356 7 -200.43 415.05 0.95 0.25

12346 7 -200.95 416.09 1.99 0.15

123456 8 -200.35 416.95 2.85 0.10

1235 6 -202.54 417.23 3.12 0.08

12345 7 -202.53 419.25 5.14 0.03

Term codes:

c.age2 c.region z.bryocov z.decomp z.height z.vedarea

1 2 3 4 5 6

Model-averaged coefficients:

Estimate Std. Error z value Pr(>|z|)

(Intercept) -1.48536 0.12104 12.271 < 2e-16 ***

c.age2 2.78819 0.27219 10.243 < 2e-16 ***

c.region 0.81810 0.12983 6.302 < 2e-16 ***

z.bryocov -0.38065 0.12962 2.937 0.00332 **

z.vedarea 0.36238 0.13417 2.701 0.00692 **

z.height 0.20577 0.16226 1.268 0.20474

z.decomp 0.07528 0.25934 0.290 0.77160

---

Signif. codes: 0 ‘***’ 0.001 ‘**’ 0.01 ‘*’ 0.05 ‘.’ 0.1 ‘ ’ 1

Relative variable importance:

c.age2 c.region z.bryocov z.vedarea z.height z.decomp

1.00 1.00 1.00 0.89 0.46 0.27

2.5 % 97.5 %

(Intercept) -1.72260434 -1.2481232

c.age2 2.25469463 3.3216756

c.region 0.56364466 1.0725509

z.bryocov -0.63469903 -0.1266032

z.vedarea 0.09941139 0.6253514

z.height -0.11225268 0.5237948

z.decomp -0.43302240 0.5835876

**Response variable: Presence/absence of Xylographa vitiligo**

**Component models:**

**df logLik AICc Delta Weight**

**125 5 -84.40 178.90 0.00 0.35**

**1256 6 -84.22 180.58 1.68 0.15**

**1235 6 -84.22 180.58 1.69 0.15**

**1245 6 -84.38 180.91 2.01 0.13**

**12356 7 -83.81 181.81 2.92 0.08**

**12345 7 -84.18 182.55 3.66 0.06**

**12456 7 -84.21 182.62 3.73 0.05**

**123456 8 -83.80 183.85 4.95 0.03**

**Term codes:**

**c.age2 c.region z.bryocov z.decomp z.height z.vedarea**

**1 2 3 4 5 6**

**Model-averaged coefficients:**

**Estimate Std. Error z value Pr(>|z|)**

**(Intercept) -4.5947 0.5285 8.694 < 2e-16 *****

**c.age2 3.0435 0.8715 3.492 0.000479 *****

**c.region 2.9134 0.7508 3.881 0.000104 *****

**z.height 1.5412 0.4227 3.646 0.000266 *****

**z.vedarea 0.3337 0.4789 0.697 0.485826**

**z.bryocov 0.3011 0.4254 0.708 0.479040**

**z.decomp -0.1627 0.9467 0.172 0.863582**

**---**

**Signif. codes: 0 ‘***’ 0.001 ‘**’ 0.01 ‘*’ 0.05 ‘.’ 0.1 ‘ ’ 1**

**Relative variable importance:**

**c.age2 c.region z.height z.bryocov z.vedarea z.decomp**

**1.00 1.00 1.00 0.32 0.32 0.27**

**2.5 % 97.5 %**

**(Intercept) -5.6304672 -3.558878**

**c.age2 1.3353479 4.751739**

**c.region 1.4419653 4.384869**

**z.height 0.7127398 2.369573**

**z.vedarea -0.6047970 1.272289**

**z.bryocov -0.5325974 1.134773**

**z.decomp -2.0180551 1.692754**

**Response variable: Presence/absence of Micarea denigrata**

Component models:

df logLik AICc Delta Weight

1236 6 -111.65 235.45 0.00 0.20

136 5 -113.45 237.01 1.56 0.09

12346 7 -111.53 237.25 1.80 0.08

12356 7 -111.58 237.35 1.90 0.08

1235 6 -112.61 237.37 1.92 0.08

123 5 -113.80 237.70 2.25 0.06

126 5 -114.04 238.19 2.74 0.05

13 4 -115.30 238.67 3.22 0.04

1234 6 -113.33 238.80 3.35 0.04

1356 6 -113.45 239.05 3.59 0.03

1346 6 -113.45 239.05 3.60 0.03

12345 7 -112.44 239.08 3.62 0.03

135 5 -114.54 239.18 3.73 0.03

123456 8 -111.48 239.21 3.76 0.03

16 4 -115.64 239.34 3.89 0.03

1246 6 -113.85 239.85 4.39 0.02

1256 6 -114.00 240.15 4.69 0.02

134 5 -115.20 240.51 5.06 0.02

13456 7 -113.45 241.10 5.64 0.01

1345 6 -114.53 241.21 5.76 0.01

146 5 -115.62 241.35 5.90 0.01

156 5 -115.63 241.38 5.92 0.01

Term codes:

c.age2 c.region z.bryocov z.decomp z.height z.vedarea

1 2 3 4 5 6

Model-averaged coefficients:

Estimate Std. Error z value Pr(>|z|)

(Intercept) -3.9506 0.5298 7.457 < 2e-16 ***

c.age2 4.2841 1.1318 3.785 0.000154 ***

c.region -0.7342 0.3826 1.919 0.054957 .

z.bryocov -0.8773 0.3944 2.224 0.026124 *

z.vedarea 0.6896 0.3627 1.901 0.057295 .

z.decomp -0.3303 0.7451 0.443 0.657529

z.height 0.2871 0.4446 0.646 0.518426

---

Signif. codes: 0 ‘***’ 0.001 ‘**’ 0.01 ‘*’ 0.05 ‘.’ 0.1 ‘ ’ 1

Relative variable importance:

c.age2 z.bryocov z.vedarea c.region z.height z.decomp

1.00 0.86 0.69 0.69 0.33 0.28

2.5 % 97.5 %

(Intercept) -4.98893295 -2.91219010

c.age2 2.06575055 6.50237781

c.region -1.48401150 0.01558719

z.bryocov -1.65028996 -0.10427862

z.vedarea -0.02136824 1.40051989

z.decomp -1.79061013 1.12999372

z.height -0.58432777 1.15858760

**Response variable: Presence/absence of Xylographa parallela**

Component models:

df logLik AICc Delta Weight

1236 6 -154.67 321.48 0.00 0.54

12356 7 -154.65 323.49 2.01 0.20

12346 7 -154.65 323.49 2.01 0.20

123456 8 -154.62 325.49 4.02 0.07

Term codes:

c.age2 c.region z.bryocov z.decomp z.height z.vedarea

1 2 3 4 5 6

Model-averaged coefficients:

Estimate Std. Error z value Pr(>|z|)

(Intercept) -3.0483 0.3722 8.191 < 2e-16 ***

c.age2 5.4781 0.7923 6.914 < 2e-16 ***

c.region 2.4067 0.3139 7.667 < 2e-16 ***

z.bryocov -0.8265 0.2896 2.854 0.004314 **

z.vedarea 1.2573 0.3399 3.699 0.000217 ***

z.height -0.0813 0.3938 0.206 0.836427

z.decomp -0.1214 0.5930 0.205 0.837844

---

Signif. codes: 0 ‘***’ 0.001 ‘**’ 0.01 ‘*’ 0.05 ‘.’ 0.1 ‘ ’ 1

Relative variable importance:

c.age2 c.region z.bryocov z.vedarea z.height z.decomp

1.00 1.00 1.00 1.00 0.27 0.27

2.5 % 97.5 %

(Intercept) -3.7776874 -2.3188537

c.age2 3.9251270 7.0310327

c.region 1.7914642 3.0220124

z.bryocov -1.3939965 -0.2589519

z.vedarea 0.5910375 1.9234744

z.height -0.8530587 0.6904617

z.decomp -1.2836939 1.0409619

**Response variable: Presence/absence of Mycocalicium subtile**

**Component models:**

**df logLik AICc Delta Weight**

**13 4 -111.99 232.04 0.00 0.11**

**123 5 -111.20 232.51 0.47 0.09**

**34 4 -112.31 232.69 0.64 0.08**

**136 5 -111.32 232.74 0.70 0.08**

**234 5 -111.38 232.86 0.82 0.07**

**1236 6 -110.61 233.36 1.32 0.06**

**134 5 -111.77 233.64 1.60 0.05**

**1234 6 -110.91 233.97 1.92 0.04**

**135 5 -111.99 234.08 2.04 0.04**

**346 5 -112.01 234.12 2.08 0.04**

**1356 6 -111.06 234.28 2.23 0.04**

**2346 6 -111.16 234.47 2.43 0.03**

**1235 6 -111.20 234.55 2.51 0.03**

**1346 6 -111.23 234.60 2.56 0.03**

**345 5 -112.25 234.61 2.57 0.03**

**2345 6 -111.33 234.81 2.77 0.03**

**12356 7 -110.41 235.03 2.98 0.02**

**12346 7 -110.47 235.13 3.09 0.02**

**3456 6 -111.66 235.48 3.43 0.02**

**1345 6 -111.76 235.67 3.63 0.02**

**23456 7 -110.88 235.96 3.92 0.02**

**12345 7 -110.90 236.01 3.96 0.02**

**13456 7 -110.94 236.07 4.03 0.01**

**123456 8 -110.24 236.73 4.69 0.01**

**3 3 -115.68 237.41 5.37 0.01**

**1 3 -115.87 237.79 5.74 0.01**

**4 3 -116.00 238.03 5.99 0.01**

**Term codes:**

**c.age2 c.region z.bryocov z.decomp z.height z.vedarea**

**1 2 3 4 5 6**

**Model-averaged coefficients:**

**Estimate Std. Error z value Pr(>|z|)**

**(Intercept) -3.4767 0.2902 11.981 <2e-16 *****

**c.age2 1.3843 0.7676 1.804 0.0713 .**

**z.bryocov -1.4827 0.5962 2.487 0.0129 ***

**c.region 0.6498 0.5263 1.235 0.2170**

**z.decomp 1.0840 0.8407 1.289 0.1973**

**z.vedarea -0.4750 0.4804 0.989 0.3227**

**z.height 0.1736 0.4741 0.366 0.7143**

**---**

**Signif. codes: 0 ‘***’ 0.001 ‘**’ 0.01 ‘*’ 0.05 ‘.’ 0.1 ‘ ’ 1**

**Relative variable importance:**

**z.bryocov c.age2 z.decomp c.region z.vedarea z.height**

**0.99 0.67 0.52 0.44 0.38 0.28**

**2.5 % 97.5 %**

**(Intercept) -4.0454228 -2.9079230**

**c.age2 -0.1200770 2.8887151**

**z.bryocov -2.6511833 -0.3142284**

**c.region -0.3817440 1.6814273**

**z.decomp -0.5637343 2.7317259**

**z.vedarea -1.4165611 0.4664974**

**z.height -0.7556805 1.1027955**

**(C) Table of explained deviance (‘pseudo-R^2^’) for stand-level GLM:s**

**Values are given for full (global) models.**

| **Response** | **Expl. Deviance** |
| --- | --- |
| **Total lichen species richness** | **0.657** |
| **Species richness of OLL** | **0.086** |
| **Micarea denigrata** | **0.607** |
| **Mycocalicium subtile** | **0.124** |
| **Xylographa parallela** | **0.823** |
| **Xylographa vitiligo** | **0.793** |

**(D) Stand-level GLM:s – output from multimodel inference in R**

Explanatory variables

<name of expl.var in this document> = <name of expl.var in main paper>

age2 = Stump age

region = Study area

X020_1000 = Buffer 0-20 years

X090_1000 = Buffer >90 years

Formulas used:

glm(<response> ~ age2 + region + X020_1000 + X90_1000,

family = binomial)

glm(<response> ~ age2 + region + X020_1000 + X90_1000,

family = poisson)

**Response variable: Total lichen species richness**

Component models:

df logLik AICc Delta Weight

123 4 -154.57 318.07 0.00 0.47

12 3 -156.56 319.66 1.59 0.21

1234 5 -154.47 320.37 2.30 0.15

124 4 -156.55 322.03 3.96 0.07

134 4 -156.86 322.64 4.57 0.05

13 3 -158.07 322.68 4.61 0.05

Term codes:

c.age2 c.region z.X020_1000 z.X90_1000

1 2 3 4

Model-averaged coefficients:

Estimate Std. Error Adjusted SE z value Pr(>|z|)

(Intercept) 3.05594 0.03241 0.03332 91.704 <2e-16 ***

c.age2 0.71020 0.06559 0.06742 10.534 <2e-16 ***

c.region 0.18186 0.06889 0.07064 2.575 0.010 *

z.X020_1000 0.13986 0.06832 0.07008 1.996 0.046 *

z.X90_1000 0.03181 0.07179 0.07343 0.433 0.665

---

Signif. codes: 0 ‘***’ 0.001 ‘**’ 0.01 ‘*’ 0.05 ‘.’ 0.1 ‘ ’ 1

Relative variable importance:

c.age2 c.region z.X020_1000 z.X90_1000

1.00 0.90 0.72 0.26

2.5 % 97.5 %

(Intercept) 2.990622382 3.1212492

c.age2 0.578058347 0.8423331

c.region 0.043409482 0.3203041

z.X020_1000 0.002508687 0.2772157

z.X90_1000 -0.112107142 0.1757292

**Response variable: Species richness of obligately lignicolous lichens**

**Component models:**

**df logLik AICc Delta Weight**

**1 2 -106.71 217.69 0.00 0.25**

**13 3 -105.68 217.90 0.21 0.22**

**123 4 -104.69 218.32 0.62 0.18**

**12 3 -106.38 219.30 1.61 0.11**

**14 3 -106.71 219.96 2.27 0.08**

**134 4 -105.68 220.29 2.60 0.07**

**1234 5 -104.52 220.46 2.77 0.06**

**124 4 -106.34 221.60 3.91 0.03**

**Term codes:**

**c.age2 c.region z.X020_1000 z.X90_1000**

**1 2 3 4**

**Model-averaged coefficients:**

**Estimate Std. Error Adjusted SE z value Pr(>|z|)**

**(Intercept) 0.84306 0.09876 0.10149 8.307 < 2e-16 *****

**c.age2 0.78209 0.19814 0.20363 3.841 0.000123 *****

**z.X020_1000 0.31574 0.19909 0.20429 1.546 0.122203**

**c.region -0.23745 0.20617 0.21140 1.123 0.261349**

**z.X90_1000 0.03336 0.19160 0.19660 0.170 0.865244**

**---**

**Signif. codes: 0 ‘***’ 0.001 ‘**’ 0.01 ‘*’ 0.05 ‘.’ 0.1 ‘ ’ 1**

**Relative variable importance:**

**c.age2 z.X020_1000 c.region z.X90_1000**

**1.00 0.53 0.39 0.24**

**2.5 % 97.5 %**

**(Intercept) 0.64414945 1.0419789**

**c.age2 0.38298857 1.1811922**

**z.X020_1000 -0.08465067 0.7161360**

**c.region -0.65178009 0.1768895**

**z.X90_1000 -0.35196907 0.4186967**

**Response variable: Occupancy of Micarea denigrata**

Component models:

df logLik AICc Delta Weight

12 3 -41.41 89.37 0.00 0.26

123 4 -40.24 89.42 0.04 0.25

1 2 -43.21 90.70 1.32 0.13

1234 5 -39.74 90.92 1.54 0.12

124 4 -41.01 90.96 1.59 0.12

13 3 -42.80 92.15 2.78 0.06

14 3 -43.21 92.97 3.60 0.04

134 4 -42.80 94.53 5.16 0.02

Term codes:

c.age2 c.region z.X020_1000 z.X90_1000

1 2 3 4

Model-averaged coefficients:

Estimate Std. Error Adjusted SE z value Pr(>|z|)

(Intercept) -3.8177 0.5116 0.5259 7.260 < 2e-16 ***

c.age2 3.7637 1.0190 1.0475 3.593 0.000327 ***

c.region -0.7960 0.3932 0.4035 1.973 0.048509 *

z.X020_1000 0.5794 0.4170 0.4280 1.354 0.175761

z.X90_1000 0.2451 0.3401 0.3484 0.703 0.481782

---

Signif. codes: 0 ‘***’ 0.001 ‘**’ 0.01 ‘*’ 0.05 ‘.’ 0.1 ‘ ’ 1

Relative variable importance:

c.age2 c.region z.X020_1000 z.X90_1000

1.00 0.74 0.45 0.30

2.5 % 97.5 %

(Intercept) -4.8484123 -2.787026552

c.age2 1.7106578 5.816694838

c.region -1.5867439 -0.005213407

z.X020_1000 -0.2593659 1.418239094

z.X90_1000 -0.4378106 0.928008904

**Response variable: Occupancy of Mycocalicium subtile**

Component models:

df logLik AICc Delta Weight

4 2 -52.55 109.36 0.00 0.26

14 3 -51.77 110.09 0.73 0.18

34 3 -52.40 111.34 1.98 0.09

24 3 -52.48 111.51 2.15 0.09

1 2 -53.82 111.91 2.55 0.07

134 4 -51.63 112.18 2.82 0.06

124 4 -51.63 112.19 2.83 0.06

12 3 -52.97 112.48 3.12 0.05

234 4 -52.37 113.67 4.31 0.03

(Null) 1 -55.91 113.90 4.54 0.03

13 3 -53.78 114.11 4.75 0.02

2 2 -55.05 114.37 5.01 0.02

1234 5 -51.55 114.52 5.16 0.02

123 4 -52.95 114.84 5.48 0.02

Term codes:

c.age2 c.region z.X020_1000 z.X90_1000

1 2 3 4

Model-averaged coefficients:

Estimate Std. Error Adjusted SE z value Pr(>|z|)

(Intercept) -2.9563 0.2005 0.2060 14.354 <2e-16 ***

z.X90_1000 0.7444 0.3237 0.3320 2.242 0.0249 *

c.age2 0.6124 0.4306 0.4416 1.387 0.1656

z.X020_1000 0.1738 0.3887 0.3993 0.435 0.6633

c.region 0.2687 0.4366 0.4472 0.601 0.5480

---

Signif. codes: 0 ‘***’ 0.001 ‘**’ 0.01 ‘*’ 0.05 ‘.’ 0.1 ‘ ’ 1

Relative variable importance:

z.X90_1000 c.age2 c.region z.X020_1000

0.79 0.49 0.29 0.25

2.5 % 97.5 %

(Intercept) -3.35997330 -2.5526586

z.X90_1000 0.09375162 1.3951299

c.age2 -0.25320235 1.4779750

z.X020_1000 -0.60874103 0.9564155

c.region -0.60790331 1.1452062

**Response variable: Occupancy of Xylographa parallela**

**Component models:**

**df logLik AICc Delta Weight**

**124 4 -53.95 116.82 0.00 0.34**

**12 3 -55.14 116.83 0.01 0.34**

**1234 5 -53.26 117.94 1.12 0.20**

**123 4 -55.02 118.96 2.14 0.12**

**Term codes:**

**c.age2 c.region z.X020_1000 z.X90_1000**

**1 2 3 4**

**Model-averaged coefficients:**

**Estimate Std. Error Adjusted SE z value Pr(>|z|)**

**(Intercept) -2.9907 0.3686 0.3789 7.892 <2e-16 *****

**c.age2 4.6170 0.7319 0.7523 6.137 <2e-16 *****

**c.region 1.7367 0.3074 0.3150 5.513 <2e-16 *****

**z.X90_1000 0.4071 0.2483 0.2551 1.596 0.111**

**z.X020_1000 0.2734 0.3107 0.3186 0.858 0.391**

**---**

**Signif. codes: 0 ‘***’ 0.001 ‘**’ 0.01 ‘*’ 0.05 ‘.’ 0.1 ‘ ’ 1**

**Relative variable importance:**

**c.age2 c.region z.X90_1000 z.X020_1000**

**1.00 1.00 0.54 0.31**

**2.5 % 97.5 %**

**(Intercept) -3.73342297 -2.2479696**

**c.age2 3.14246614 6.0914646**

**c.region 1.11932052 2.3540730**

**z.X90_1000 -0.09288111 0.9071457**

**z.X020_1000 -0.35107229 0.8979100**

**Response variable: Occupancy of Xylographa vitiligo**

Component models:

df logLik AICc Delta Weight

124 4 -28.64 66.21 0.00 0.50

1234 5 -27.49 66.41 0.20 0.45

12 3 -32.21 70.97 4.76 0.05

Term codes:

c.age2 c.region z.X020_1000 z.X90_1000

1 2 3 4

Model-averaged coefficients:

Estimate Std. Error Adjusted SE z value Pr(>|z|)

(Intercept) -4.4178 0.5224 0.5368 8.230 < 2e-16 ***

c.age2 2.5515 0.7620 0.7834 3.257 0.00113 **

c.region 2.5030 0.7603 0.7815 3.203 0.00136 **

z.X90_1000 1.0243 0.4141 0.4232 2.420 0.01550 *

z.X020_1000 0.7446 0.4883 0.5024 1.482 0.13833

---

Signif. codes: 0 ‘***’ 0.001 ‘**’ 0.01 ‘*’ 0.05 ‘.’ 0.1 ‘ ’ 1

Relative variable importance:

c.age2 c.region z.X90_1000 z.X020_1000

1.00 1.00 0.95 0.45

2.5 % 97.5 %

(Intercept) -5.4699270 -3.365638

c.age2 1.0159547 4.087011

c.region 0.9712753 4.034759

z.X90_1000 0.1948376 1.853710

z.X020_1000 -0.2401297 1.729399
